# Assessment of the Refractive Error and Stabilisation of Refraction after Cataract Surgery in Relation to the Length of the Eyeball

**DOI:** 10.3390/jcm11185447

**Published:** 2022-09-16

**Authors:** Małgorzata Mrugacz, Mateusz Olszewski, Magdalena Pony-Uram, Jacek Brymerski, Anna Bryl

**Affiliations:** 1 Department of Ophthalmology and Eye Rehabilitation, Medical University of Bialystok, Waszyngtona 17, 15-274 Bialystok, Poland; 2 Department of Ophthalmology, Subcarpathian Hospital in Krosno, Korczynska 57, 38-400 Krosno, Poland

**Keywords:** cataract, cataract surgery, intraocular lens, refractive error, refraction

## Abstract

The aim of this study was to analyse the changes in refraction, depending on the length of the eyeball, in patients who had undergone cataract surgery using the phacoemulsification method and to assess the stability of refraction. A total of 90 patients (46 to 85 years of age) took part in the study and were divided into three groups: emmetropic, hypermetropic, and myopic. Two types of intraocular lenses were used: Bausch (Akreos AO) and Rayner (C-flex). In conclusion, stabilization of refraction was achieved in the third week in 91% of the emmetropic, 77% of the myopic, and 46% of the hypermetropic patients, respectively. The correct postoperative refraction was achieved using optical biometry and the Barrett Universal II formula to calculate the power of the lens implant.

## 1. Introduction

Next to refractive errors, cataracts are the most common cause of the deterioration of vision in the world, resulting in cataract surgery becoming the most common surgical procedure performed in developed countries [1]. As the standards of surgical treatment increase, so do the societal expectations. Achieving the target refraction with surgery is currently a priority for both the patient and the surgeon, and the surgery itself is considered to be a refractive procedure.

The postoperative refractive error caused by this operation is a major clinical concern because it directly affects a patient’s life quality. However, after cataract surgery, the refractive error in approximately 5% to 20% patients is greater than 1 diopter [2,3].

The axial length of the eye is one of the basic parameters determining the power of the implanted lens. Errors in the calculations of biometry cause 54% of the errors in refraction after phacoemulsification [4].

We hypothesized that refraction after cataract surgery using the phacoemulsification method and its stabilization time are dependent on the length of the eyeball.

## 2. Material and Methods

A total of 90 patients (29 men and 61 women) 46 to 85 years of age took part in the study. The mean age of the participants was 71.63 years. All patients suffered from age-related cataracts which made them eligible for phacoemulsification treatment. Patients with diseases of the anterior segment of the eyeball, e.g., pterygium, corneal scars, a history of eye injury, myopia with significant changes in the central part of the retina, and diseases of the optic nerve after vitrectomy with intra-operative and postoperative complications, were excluded from the study. The cataracts were removed by phacoemulsification with implantation of artificial lenses in all patients participating in the study. Bausch (Akreos AO) and Rayner (C-flex) intraocular lenses were implanted. The difference between the lenses is the number of haptics—the Akreos has four small haptics and the C-flex has two longer ones on opposite sides. The aim of using the different lenses was to analyse the influence of the haptic number on the refractive errors after cataract surgery. The Barrett Universal II formula was used for the calculations. The length of the eyeball, the depth of the anterior chamber, and the thickness of the cornea were measured using a Lenstar 900 apparatus. All of these tests were performed after pupil dilation.

Referring to the schematic model of the Gullstrand eye, it can be assumed that the axial length of the eyeball is, on average, 24.4 mm. Considering the correlation between refractive error and axial length, the patients in our study were divided into the following groups:

Group I consisted of 30 people suffering from cataracts, with emmetropic vision, with an eye length in the range of 22–24 mm (21 women and 9 men in the range of 46–83 years of age, with an average age of 71 years).

Group II consisted of 30 people suffering from cataracts, with hypermetropic vision, with an eye length of less than 22 mm (24 women and 6 men in the age range of 55–85 years, with an average age of 74 years).

Group III consisted of 30 people suffering from cataracts, with myopic vision, with an eye length of more than 24 mm (16 women and 14 men in the age range of 48–83 years, with an average age of 72.5 years).

**Figure 1:**
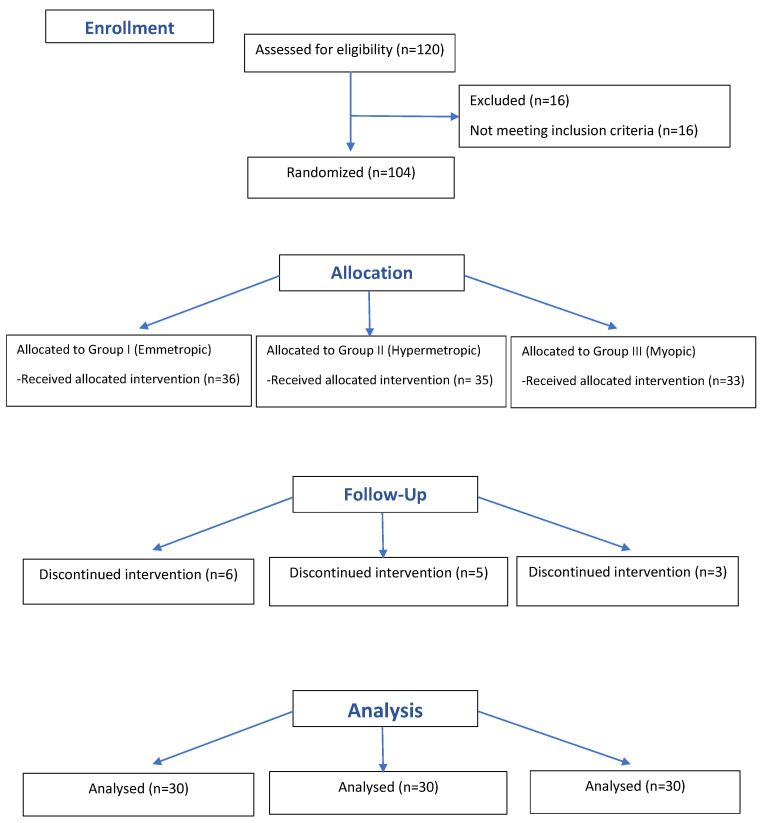
Consort flow diagram of the study.

The consort flow diagram Figure 1 presents study recruitment. 

A complete ophthalmological examination was performed on all patients before the procedure and in the third week and the third month following the procedure.

Visual acuity (visus) was tested with Snellen charts, assessing both far (5 m) and near (40 cm) vision, with the best possible vision correction using glasses. Objective refraction was tested using a Topcon autoceratorefractometer (KR-8800). The autorefractor result was the mean of the third measurement for each patient. 

All research was conducted in accordance with the principles set out in the Helsinki Declaration and after obtaining the consent of the bioethics committee of the Medical University of Bialystok.

## 3. Statistical Analysis

The chi-square test was used to compare the primary qualitative variable between the three groups (emmetropic, hypermetropic, and myopic patients) and to compare two other qualitative variables, or in the case of failure, to meet the assumptions of the expected counts, using a nonparametric counterpart-Fisher’s exact test. In quantitative analysis, the Kruskal–Wallis test was used to compare the three groups of patients since the lack of a normal distribution of the data in the individual groups was demonstrated with the Shapiro-Wilk test. To compare the same primary variable, measured at three time points (0 (before surgery), 3 weeks after surgery, and 3 months after surgery), a non-parametric Friedman test was used. 

A *p* value of ≤0.05 was considered statistically significant. The statistical analysis was performed with R software version 4.0.0 (https://www.r-project.org/, (accessed on 12 September 2022)).

## 4. Results

The study group consisted of people between 46 and 85 years of age. The median age ranged from 71 to 74 years. The demographic differences between the groups were not statistically significant (Table 1).

### Analysis of Refraction Changes following Cataract Surgery

The absolute error, i.e., the absolute value of the individual deviations, was analysed. The deviations constituted the differences between the actual postoperative spherical equivalent in objective examination (SE 3 m) and the assumed refractive result before surgery (planned SE). The absolute error was adopted as a parameter that better described the refractive result of the procedure than the postoperative refraction because, in the latter case, the negative and positive values cancelled each other out.

Table 2 shows all the observed cases of change in the planned SE values compared to the SE values measured in the third month.

The characteristics of the analysed groups for the SE parameter are summarized in Table 3.

**Figure 2:**
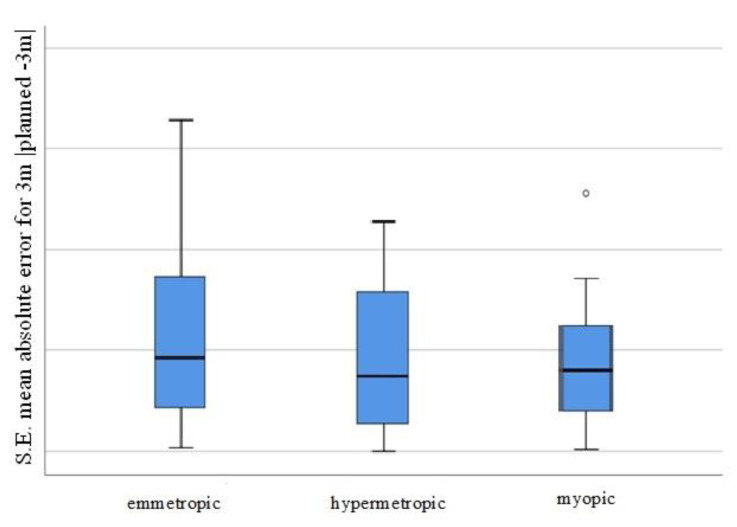
Visual representation of the MAE for the individual patient groups (SE mean absolute error for 3 m, planned −3 m, for the emmetropic/hypermetropic/myopic groups).

MAE representation for the tree groups (emmetropic, hypermetropic, myopic) is presented on Figure 2.

When analysing the values of the absolute errors after the procedures, no differences were found between the three groups (hypermetropic, emmetropic, and myopic) at 3 weeks and at 3 months. Further, there were no differences between the absolute errors for the three-week and the three-month periods in each of the three groups (hypermetropic, emmetropic, and myopic) (all *p* values were >0.1).

The SE mean absolute errors (3 months vs. planning) were converted in two ways:

▪ Mean absolute error (MAE) classification:
  - 0 for MAE_SE of <0.25 (29 people, 32%)
  - 1 for MAE_SE from 0.25 to 0.5 (29 people, 32%)
  - 2 for MAE_SE from 0.5 to 0.75 (10 people, 11%)
  - 3 for MAE_SE from 0.75 to 1 (11 people, 12%)
  - 4 for MAE_SE of 1 and higher (11 people, 12%)
▪ Absolute error (SE_change) classification:
  −1 if, after 3 months, the SE value decreased from the planned SE (67 people)
  - 0 if, after 3 months, the SE value was the same as the planned SE (1 person)
  - +1 if, after 3 months, the SE value increased above the planned SE (22 people)

The MAE classification is reported in Table 4.

Table 5 shows the SE changes (3 t–3 m) in the analysed groups.

## 5. Discussion

In the analysed material, the mean of the best correction of distance acuity and near vision acuity was achieved at the third week and at the third month after the procedure, with values of 1.0 and 0.5, respectively, on the Snellen scale. Refraction was assessed by comparing the spherical equivalent parameter with the use of an autoceratorefractometer at the third week and at the third month.

The stabilization of refraction within the bounds of +/− 0.5 spherical D in the third week was achieved in 91% of the emmetropic, 77% of the myopic, and 46% of the hypermetropic patients, respectively. The percentage distribution within the limits of +/− 0.75 spherical D was as follows: 94% in the emmetropic group, 94% in the myopic group, and 80% in the hypermetropic group. The fastest refraction stabilization occurred in the emmetropic group, as the change of the spherical equivalent lower than 0.25 spherical D was achieved in 57% of the participants (*p* < 0.003). We speculate that the extended stabilization time in myopic patients was related to more relaxed lens zonules, larger vitreous cavities, and vitreous liquefaction. On the other hand, hypermetropic eyes have a small capsular diameter that may result in a bending of the optic-haptic junction during the postoperative period, which could then normalize over time.

Many publications have attempted to determine the period of refraction stabilization after the procedure, and thus, to determine the most optimal period for prescribing corrective glasses for a patient [5,6,7,8,9]. One such study was conducted by Berka et al., who examined 1838 eyes subjected to phacoemulsification manually and using a femtosecond laser [10]. Visual acuity and refraction stabilized, as measured by both of these methods, 3 weeks after the procedure. In the study by Sugar et al., stabilization of refraction (spherical equivalent, cylinder, and cylinder axis) was achieved at the end of 1 week [11]. Similar results were obtained by Juan et al., who determined that stabilization of refraction was achieved at the end of 1 week after phacoemulsification surgery [12]. The data from the literature are consistent with the data obtained in our study regarding the group of emmetropic patients, with 94% achieving refractive stabilization within +/− 0.5 Dsph 3 weeks after the procedure. In the groups of hypermetropic and myopic patients, the difference was slightly greater, and was within a similar percentage in the range of +/− 0.75 Dsph. The largest observed difference in spherical equivalent between the third week and the third month was 1.5 Dsph. The lack of control points between the third week and the third month after surgery and the use of two lens models with different types of haptics (which may have affected the lens stabilization period after surgery) were limitations of the study. Our research indicated that the third week, which was the first follow-up test point, should be considered safe in terms of prescribing corrective glasses to emmetropic patients. However, the patients should always be informed of the small likelihood of a change in lens power after a few weeks.

Based on our research, the refractive result achieved after cataract surgery was analysed. The absolute errors, i.e., the absolute values of the individual deviations, were assessed. The obtained MAEs were 0.47 D for emmetropic eyes, 0.38 D for hypermetropic eyes, and 0.41 D for myopic eyes. There were no statistically significant differences between the study groups (*p* = 0.503). The MAE for the entire study population was 0.42 D, and 64% of the patients achieved target refraction within the MAE range up to 0.5 D and 88% achieved it in the 1D range.

The choice of an implant lens depends on the use of an appropriate formula. A formula that is equally accurate and reliable for the length range of each eyeball has been sought for many years. The most modern formulas available in the market are the fourth-generation formulas, which include the Barrett Universal II, Olsen, and Holladay 2 formulas.

The Barrett Universal II formula was introduced in 2010 by Graham D Barrett [13]. It appears to be a universal formula and is equally effective in each of the studied groups.

When analysing the relevant literature, it is worth noting that errors that may appear in the groups of patients with elongated eyeballs may not always be the result of errors in the calculation formula, but rather, they may result from the use of contact ultrasound biometry. The use of this type of measuring equipment in extremely elongated eyeballs with posterior staphyloma may cause a refractive error of 3–5 Dsph [14,15,16]. Another variable is the researcher. The use of contact ultrasound biometry by an inexperienced person may generate significant postoperative refractive errors. Unfortunately, it is not always possible to perform an examination with the use of optical biometry, and advanced opacities in a lens may make it impossible to perform the measurement [17]. In such a situation, the only option is to perform an examination using ultrasound biometry with a prior ultrasound examination of the posterior segment in order to rule out posterior staphyloma [18].

## 6. Conclusions

Refraction after cataract surgery stabilizes faster in emmetropic eyes than in myopic or hypermetropic eyes. This result shows that in this group of patients, the third week post-surgery is the earliest time to prescribe reading glasses.

## Figures and Tables

**Table 1:** Characteristics of the analysed group in terms of demographic data and treatment method.

	Emmetropic Patients (1) *n* = 30	Hypermetropic Patients (2) *n* = 30	Myopic Patients (3) *n* = 30	*p*
Sex				0.083
Women	70% (21)	80% (24)	53% (16)	
Men	30% (9)	20% (6)	47% (14)	
Lens				0.036
R	73% (22)	83% (25)	53% (16)	
B	27% (8)	17% (5)	47% (14)	
Age	71.0 (66; 78) 46; 83	74.0 (70; 80) 55; 85	72.5 (66; 75) 48; 83	0.730

Qualitative variables: percentage (number of people), B—Bausch (Akreos AO), and R—Rayner (C-flex).

**Table 2:** The differences in the planned spherical equivalents against the spherical equivalents measured in the third month post-surgery (number of people).

	Emmetropic Patients (1) *n* = 30	Hypermetropic Patients (2) *n* = 30	Myopic Patients (3) *n* = 30
The value decreased	76% (23)	56% (17)	90% (27)
The value increased to 0	10% (3)	10% (3)	0
The value has increased and became positive	4% (1)	10% (3)	4% (1)
The value has increased but was still negative	10% (3)	20% (6)	6% (2)
The value remained the same	0	4% (1)	0

**Table 3:** The SE value characteristics of the analysed groups.

	Emmetropic Patients (1) *n* = 30	Hypermetropic Patients (2) *n* = 30	Myopic Patients (3) *n* = 30	*p*
Planned SE	−0.23 (−0.35; −0.14) −0.48; 0	−0.24 (−0.32; −0.16) −0.43; −0.11	−0.38 (−2.59; −0.18) −2.88; −0.12	0.001 1 vs. 3, 2 vs. 3
SE before surgery	0.94 (−0.41; 1.37) −9.25; 3.37	2.25 (0.59; 4.03) −1.75; 9	−3.12 (−7.21; −1.16) −15.87; −0.62	<0.001 1 vs. 2, 2 vs. 3, 1 vs. 3
SE 3 weeks after surgery	−0.56 (−1; −0.37) −2.25; 0.87	−0.62 (−1; 0) −1.62; 0.62	−1 (−2.87; −0.5) −4.5; 0.5	0.015 2 vs. 3, 1 vs. 3
SE 3 months after surgery	−0.63 (−1; −0.25) −1.75; 1	−0.44 (−0.75; −0.12) −1.5; 0.87	−0.75 (−3; −0.5) −4; 0.25	0.004 2 vs. 3
SE 3 m-3 w				
SE MAE_3 w	0.39 (0.22–0.73) 0–2.25	0.55 (0.25–0.82) 0.02–1.45	0.37 (0.24–0.67) 0.01–1.64	0.445
SE MAE_3 m	0.47 (0.22–0.87) 0.02–1.64	0.38 (0.14–0.79) 0–1.14	0.41 (0.20–0.62) 0.01–1.28	0.503

Quantitative variable median (first quartile, Q1; third quartile, Q3); minimum, maximum.

**Table 4:** Characteristics of the analysed groups in terms of the absolute errors after 3 months.

	Emmetropic Patients (1) *n* = 30	Hypermetropic Patients (2) *n* = 30	Myopic Patients (3) *n* = 30	*p*
MAE				
<0.25	30% (9)	37% (11)	30% (9)	0.517
[0.25–0.50]	23% (7)	30% (9)	43% (13)	
[0.50–0.75]	17% (5)	7% (2)	10% (3)	
[0.75–1]	10% (3)	13% (4)	13% (4)	
≥1	20% (6)	13% (4)	4% (1)	
SE change				
−1	77% (23)	57% (17)	90% (27)	0.024
0	-	3% (1)	-	
1	23% (7)	40% (12)	10% (3)	

**Table 5:** Characteristics of the analysed groups in terms of the SE changes (3 t–3 m).

	Emmetropic Patients (1) *n* = 30	Hypermetropic Patients (2) *n* = 30	Myopic Patients (3) *n* = 30	*p*
SE 3 t–3 m				
<0.25	57% (17)	23% (7)	34% (10)	0.003
[0.25–0.50]	34% (10)	23% (7)	43% (13)	
[0.50–0.75]	3% (1)	34% (10)	17% (5)	
[0.75–1]	3% (1)	20% (6)	3% (1)	
≥1	3% (1)	0% (0)	3% (1)	

## Data Availability

The data presented in this study are available in the article. Eventual additional data are available on request from the corresponding author.
